# Influence of polypharmacy on patients with heart failure with preserved ejection fraction: a retrospective analysis on adverse outcomes in the TOPCAT trial

**DOI:** 10.3399/bjgp21X714245

**Published:** 2020-12-01

**Authors:** Yuzhong Wu, Wengen Zhu, Xin He, Ruicong Xue, Weihao Liang, Fangfei Wei, Zexuan Wu, Yuanyuan Zhou, Dexi Wu, Jiangui He, Yugang Dong, Chen Liu

**Affiliations:** Department of Cardiology, the First Affiliated Hospital of Sun Yat-Sen University, Guangzhou 510080, PR China;; Department of Cardiology, the First Affiliated Hospital of Sun Yat-Sen University, Guangzhou 510080, PR China;; Department of Cardiology, the First Affiliated Hospital of Sun Yat-Sen University, Guangzhou 510080, PR China;; Department of Cardiology, the First Affiliated Hospital of Sun Yat-Sen University, Guangzhou 510080, PR China;; NHC Key Laboratory of Assisted Circulation (Sun Yat-Sen University), Guangzhou 510080, PR China;; NHC Key Laboratory of Assisted Circulation (Sun Yat-Sen University), Guangzhou 510080, PR China;; NHC Key Laboratory of Assisted Circulation (Sun Yat-Sen University), Guangzhou 510080, PR China;; NHC Key Laboratory of Assisted Circulation (Sun Yat-Sen University), Guangzhou 510080, PR China;; NHC Key Laboratory of Assisted Circulation (Sun Yat-Sen University), Guangzhou 510080, PR China;; NHC Key Laboratory of Assisted Circulation (Sun Yat-Sen University), Guangzhou 510080, PR China;; National-Guangdong Joint Engineering Laboratory for Diagnosis and Treatment of Vascular Diseases, Guangzhou, PR China.; Department of Cardiology, the First Affiliated Hospital of Sun Yat-Sen University, Guangzhou 510080, PR China;

**Keywords:** heart failure, hospitalisation, medication burden, outcome, patient readmission, polypharmacy

## Abstract

**Background:**

Polypharmacy is common in heart failure (HF), whereas its effect on adverse outcomes in patients with HF with preserved ejection fraction (HFpEF) is unclear.

**Aim:**

To evaluate the prevalence, prognostic impacts, and predictors of polypharmacy in HFpEF patients.

**Design and setting:**

A retrospective analysis performed on patients in the Americas region (including the US, Canada, Argentina, and Brazil) with symptomatic HF and a left ventricular ejection fraction ≥45% in the TOPCAT (Treatment of Preserved Cardiac Function Heart Failure with an Aldosterone Antagonist) trial, an international, randomised, double-blind, placebo-controlled study conducted during 2006–2013 in six countries.

**Method:**

Patients were categorised into four groups: controls (<5 medications), polypharmacy (5–9 medications), hyperpolypharmacy, (10–14 medications), and super hyperpolypharmacy (≥15 medications). The outcomes and predictors in all groups were assessed.

**Results:**

Of 1761 participants, the median age was 72 years; 37.5% were polypharmacy, 35.9% were hyperpolypharmacy, and 19.6% were super hyperpolypharmacy, leaving 7.0% having a low medication burden. In multivariable regression models, three experimental groups with a high medication burden were all associated with a reduction in all-cause death, but increased risks of HF hospitalisation and all-cause hospitalisation. Furthermore, several comorbidities (dyslipidemia, thyroid diseases, diabetes mellitus, and chronic obstructive pulmonary disease), a history of angina pectoris, diastolic blood pressure <80 mmHg, and worse heart function (the New York Heart Association functional classification level III and IV) at baseline were independently associated with a high medication burden among patients with HFpEF.

**Conclusion:**

A high prevalence of high medication burden at baseline was reported in patients with HFpEF. The high medication burden might increase the risk of hospital readmission, but not the mortality.

## INTRODUCTION

Heart failure (HF) is a complex clinical syndrome with an expected prevalence of 46% by 2030.^1^ Patients with HF are commonly affected by a series of comorbidities,^2^ and regularly prescribed with multiple medications. Current therapeutic regimens could lead to a heavy medication burden such as polypharmacy^3^ and hyperpolypharmacy.^4^ In patients with HF, a previous review^3^ has revealed that polypharmacy was an underacknowledged cause of health problems,^5^^,^^6^ and it also elucidated a detrimental impact of polypharmacy on medication compliance,^7^ drug-drug interactions,^8^ incidence of underprescription and inaccurate prescription, and drug-related adverse reactions.^9^ The most common type of HF is HF with preserved ejection fraction (HFpEF), which will have an increasing prevalence in the upcoming decades.^10^ HFpEF is typically considered as a patient having left ventricular ejection fraction (LVEF) ≥50%, whereas in trials of HFpEF, patients with HF with mid-range ejection fraction with LVEF between 40% and 49% have generally been included.^11^ HFpEF is associated with increased hospital readmission rate and mortality. However, other than patients with HF with reduced ejection fraction (HFrEF), who have a number of therapies that have been found to improve morbidity and mortality, drug treatments of HFpEF patients have no such clinical benefits.^11^ The complicated therapeutic regimens in HFpEF patients always reflect symptom alleviation and treatment for comorbidities. Until now, there has been a dearth of studies for the impact of high medication burden on clinical outcomes among patients with HFpEF. In the TOPCAT (Treatment of Preserved Cardiac Function Heart Failure with an Aldosterone Antagonist) trial,^12^ patients with HFpEF were included to:

- examine the prevalence of high medication burden;
- assess the association of high medication burden at baseline with clinical outcomes; and
- explore the predictors of high medication burden.

## METHOD

### Study design and participants

The TOPCAT trial, an international, randomised, double-blind, placebo-controlled study sponsored by the US National Heart, Lung, and Blood Institute, was established to determine the therapeutic role of spironolactone in patients with symptomatic HFpEF. The study was held from August 2006 to June 2013, reaching a mean follow-up time of 3.3 years.^13^ This trial enrolled 3445 patients from six countries who had provided written informed consents. Male and female patients had an age of ≥50 years and a LVEF of ≥45%. Each patient should have at least one hospitalisation within 12 months for which HF was a major component of the hospitalisation, or if not, have an elevated level of the natriuretic peptide within 60 days before randomisation (B-type natriuretic peptide level of ≥100 pg/ml or N-terminal pro-B-type natriuretic peptide of ≥360 pg/ml). Exclusion criteria mainly included a severe systemic illness with life expectancy of <3.0 years from randomisation, serum potassium ≥5.0 mmol/L, severe renal dysfunction (estimated glomerular filtration rate [eGFR] <30 ml/min/1.73 m^2^ of body-surface area or creatinine ≥2.5 mg/dL), treatment with aldosterone antagonists or potassium-sparing diuretics within 14 days before the randomisation, or recent acute events as described previously.^13^ The institutional review board at each of the participating sites approved the primary study protocol.

**Table:** How this fits in

High medication burden is common in the treatment of chronic diseases and has been shown to be associated with increased risks of adverse cardiovascular outcomes. This research found that high medication burden might increase the risk of hospital readmission, but not the mortality in certain patients with heart failure (HF). Risk of polypharmacy urges prescription optimisation. Clinicians may need to simplify prescriptions when patients are taking multiple drugs simultaneously.

There were significant regional differences (Americas, including US, Canada, Argentina and Brazil, versus Russia and Georgia) in the baseline characteristics of patients, outcomes, and treatment response to spironolactone in the TOPCAT trial.^12^ Consistent with most previous studies,^14^^,^^15^ only participants from the Americas were included in the present study. Six patients with missing data regarding baseline medication were excluded, resulting in a final sample size of 1761.

### Assessment of medication burden at baseline

The data on patients’ medication profiles were abstracted (for example, names of drugs, dosages, and total daily numbers). The medication information was collected based on a combination of medical record review and interview at baseline visit. All of these eligible participants were divided into four groups according to the total number of prescription medications at baseline: low medication burden (controls, defined as <5 different medications), polypharmacy (defined as 5–9 medications),^16^^,^^17^ hyperpolypharmacy (defined as 10–14 medications),^4^^,^^18^ and super hyperpolypharmacy (defined as ≥15 medications). For the 142 cases using combination medications (combo drugs), the numbers of effective constituents of these drugs were applied in calculating the individual total medications.

### Outcomes

Consistent with the TOPCAT trial, the primary outcome was a composite of cardiovascular death, aborted cardiac arrest, or HF hospitalisation. The secondary outcomes included cardiovascular death, all-cause death, HF hospitalisation, all-cause hospitalisation, myocardial infarction, and stroke. The detailed definitions of these outcomes referred to in a previous description.^13^ During the follow up, the outcomes were monitored through subject contacts and by interview and medical record review at the clinic site.

### Potential confounders

Data on the potential confounders at baseline were extracted as follows: age, sex, race, randomisation arm (spironolactone or placebo), heart rate, systolic blood pressure, diastolic blood pressure (DBP), body mass index, abdominal obesity (defined as waist circumference ≥88 cm in females and ≥102 cm in males), anaemia (defined as hemoglobin <12g/dl in females and <13g/dl in males), smoking status, New York Heart Association (NYHA) functional class, Quasi Random Signal (QRS) duration, laboratory values (eGFR, serum K^+^ and Na^+^, serum creatinine, aspartate aminotransferase, and alanine aminotransferase), and comorbidities (diabetes mellitus [DM], hypertension, dyslipidemia, chronic obstructive pulmonary disease [COPD], history of HF hospitalisation, stroke, myocardial infarction, percutaneous coronary intervention, coronary artery bypass graft surgery, atrial fibrillation, asthma, thyroid diseases, and peripheral arterial disease).

The demographic information and medical histories were collected based on a combination of medical record review and patient self-report. Smoking statuses were self-reported. Values of physical examinations and laboratory tests were acquired from actual operations.

### Statistical analysis

Baseline characteristics were summarised using frequencies and proportions for categorical variables, medians and interquartile ranges (IQRs) for continuous variables, and compared by χ^2^ tests and Wilcoxon-Mann-Whitney tests, respectively. Event rates with 95% confidence intervals (CIs) and Kaplan-Meier curves tested by the log-rank method were given to describe the outcomes for all studied groups. For the primary outcome, cardiovascular death, and all-cause death, Cox proportional hazards models were used to calculate hazard ratios (HRs). For events not related to death (hospitalisations, myocardial infarction, and stroke), competing risk regression models were applied. Very few missing values in covariates (no more than 17 values in a single covariate) were supplemented using the regression imputation method for retaining all the samples in multivariable models. Owing to the distribution of medication burden status, log-binomial regression was applied for identifying predictors of high medication burden. All potential predictors that showed significance in univariable models were included in the corresponding multivariable model. The statistical analyses were performed in R (version 3.6.1), with packages of tableone, mice, survival, survminer, cmprsk, and lbreg *.* A two-tailed *P*-value of <0.05 was considered statistically significant.

## RESULTS

### Baseline characteristics

**Figure 1.**
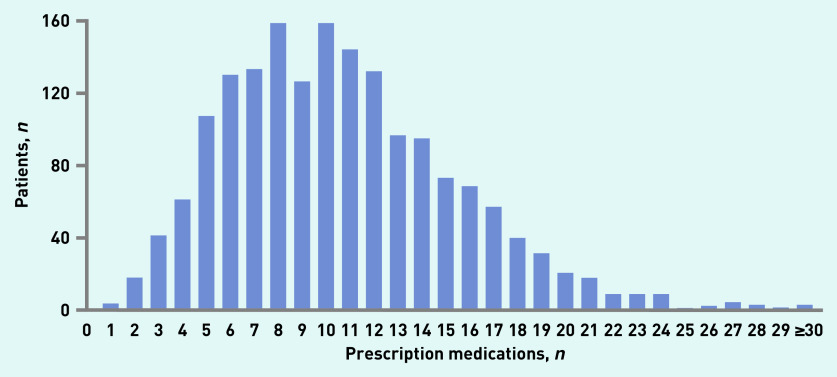
**The distribution of total medication burden at baseline in patients with heart failure with preserved ejection fraction.**

**Figure 2.**
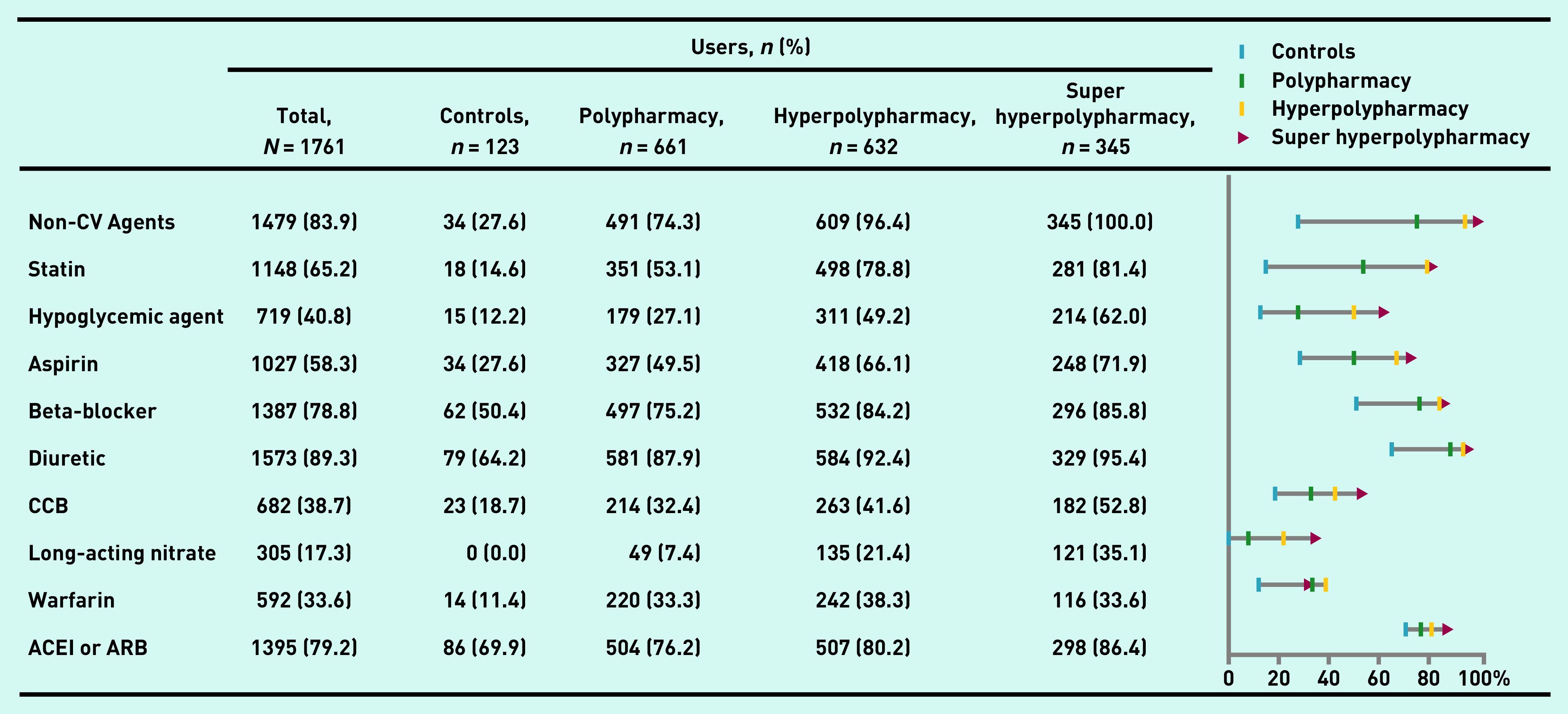
**Medication usage characteristics in different medication burden status. ACEI = angiotensin-converting enzyme inhibitor. ARB = angiotensin receptor blocker. CCB = calcium channel blocker. CV = cardiovascular.**

Of 1761 eligible patients with HFpEF, the median age was 72 years (IQR = 64–79), 42.4% were aged ≥75 years, and 49.9% were female. In total, 123 (7.0%) patients had low medication burden, whereas the prevalence of polypharmacy, hyperpolypharmacy, and super hyperpolypharmacy was 37.5%, 35.9%, and 19.6%, respectively (Figure 1). The greatest amount of increase in weight of total medication burden was for non-cardiovascular medications (Figure 2; see Supplementary Table S1 for details). As shown in Table 1, in comparison with patients with low medication burden, those with high medication burden were predisposed to obesity, anaemia, lower diastolic blood pressure, worse kidney function (lower eGFR), more comorbidities, to be smokers, and belong to NYHA functional class III/IV.

**Table 1.** Baseline characteristics of patients by total medication burden at baseline

**Characteristic^a^**	**Total *N*= 1761**	**Controls *n*= 123**	**Polypharmacy *n*= 661**	**Hyperpolypharmacy *n*= 632**	**Super hyperpolypharmacy *n*= 345**	***P*-value**
**Spironolactone**	883 (50.1)	62 (50.4)	328 (49.6)	310 (49.1)	183 (53.0)	0.67

**Age, years**						
Mean (IQR)	72 (64–79)	72 (63–79)	73 (65–80)	73 (64–80)	71 (63–78)	0.07
≥75	746 (42.4)	52 (42.3)	296 (44.8)	266 (42.1)	132 (38.3)	0.26

**Female**	879 (49.9)	67 (54.5)	344 (52.0)	301 (47.6)	167 (48.4)	0.28

**White (ethnicity)**	1378 (78.3)	94 (76.4)	506 (76.6)	510 (80.7)	268 (77.7)	0.30

**Heart rate, beats/min**	68 (61–76)	72 (65–78)	69 (60–76)	68 (61–75)	68 (60–76)	0.009

**SBP, mmHg**	129 (118–138)	130 (120–139)	128 (118–138)	130 (118–139)	126 (116–139)	0.43

**DBP, mmHg**	70 (62–80)	80 (70–82)	72 (64–80)	70 (62–80)	68 (60–76)	<0.001

**BMI, kg/m^2^**	32.9 (28.0–38.4)	30.7 (25.7–34.8)	31.6 (27.1–36.6)	33.4 (29.1–39.2)	35.3 (28.9–40.3)	<0.001

**BMI classification**						<0.001
<18.5 (underweight)	8 (0.5)	1 (0.8)	4 (0.6)	3 (0.5)	0 (0.0)	
18.5-24.9 (normal weight)	208 (11.8)	27 (21.9)	89 (13.5)	56 (8.9)	36 (10.4)	
25.0-29.9 (overweight)	408 (23.2)	29 (23.6)	186 (28.1)	129 (20.4)	64 (18.6)	
≥30 (obesity)	1137 (64.6)	66 (53.7)	382 (57.8)	444 (70.3)	245 (71.0)	

**Waist circumference, cm**	109.2 (97.0–121.9)	103.0 (93.0–114.0)	106.0 (94.0–116.8)	111.8 (100.0–121.9)	115.0 (100.0–127.0)	<0.001

**Abdominal obesity**	1394 (79.2)	85 (69.1)	501(75.8)	526 (83.2)	282 (81.7)	<0.001

**Smoking status**						<0.001
Current smoking	747 (42.4)	70 (56.9)	309 (46.7)	246 (38.9)	122 (35.4)	
Ever smoking	117 (6.6)	12 (9.8)	48 (7.3)	35 (5.5)	22 (6.4)	
Never smoking	897 (50.9)	41 (33.3)	304 (46.0)	351 (55.5)	201 (58.3)	

**QRS duration, ms**	93 (80–106)	90 (80–101)	94 (82–108)	94 (82–107)	92 (80–104)	0.41

**LVEF**	58 (53–64)	59 (55–65)	59 (50–65)	58 (54–62)	58 (54–63)	0.32

**LVEF classification**						0.006
45–54	484 (27.5)	30 (24.4)	206 (31.2)	161 (25.5)	87 (25.2)	
55–64	848 (48.2)	58 (47.2)	280 (42.4)	323 (51.1)	187 (54.2)	
≥65	429 (24.4)	35 (28.5)	175 (26.5)	148 (23.4)	71 (20.6)	

**NYHA functional class**						<0.001
I and II	1142 (64.8)	97 (78.9)	472 (71.4)	391 (61.9)	182 (52.8)	
III and IV	619 (35.2)	26 (21.1)	189 (28.6)	241 (38.1)	163 (47.2)	

**Anaemia**	722 (41.0)	29 (23.6)	227 (34.3)	281 (44.5)	185 (53.6)	<0.001

**Laboratory values**						
Haemoglobin, mg/dl	12.8 (1.7)	13.5 (1.5)	13.1 (1.7)	12.7 (1.6)	12.4 (1.6)	<0.001
WBC, k/uL	7.1 (5.9–8.5)	6.7 (5.6–8.3)	6.9 (5.9–8.3)	7.2 (6.0–8.7)	7.1 (5.9–8.7)	0.020
PLT, k/uL	219 (181–264)	224 (180–275)	218 (183–263)	218 (178–265)	219 (185–262)	0.91
Serum Na+, mg/dl	140 (138–142)	140 (138–142)	140 (138–142)	140 (138–142)	140 (138–141)	0.55
Serum K+, mg/dl	4.2 (3.9–4.5)	4.3 (3.9–4.5)	4.2 (3.9–4.5)	4.2 (3.9–4.5)	4.2 (3.8–4.4)	0.028
ALT, U/L	22 (15–31)	19 (13–27)	22 (15–31)	23 (16–32)	21 (16–31)	0.015
AST, U/L	22 (18–29)	22 (17–28)	22 (18–28)	23 (18–30)	23 (18–30)	0.07
ALP, U/L	83 (66–112)	93.9 (74–148)	84 (66–116)	82 (66–107)	82 (63–108)	<0.001
Serum creatinine, mg/dl	1.1 (0.9–1.4)	1.0 (0.8–1.2)	1.1 (0.9–1.3)	1.2 (0.9–1.4)	1.2 (1.0–1.5)	<0.001
eGFR, ml/min	61.2 (49.0–76.5)	73.5 (57.9–86.5)	64.0 (51.5–78.6)	58.7 (48.7–73.2)	56.4 (44.9–71.6)	<0.001

**Comorbidities**						
Previous HF hospitalisation	1039 (59.0)	65 (52.8)	389 (58.9)	359 (56.8)	226 (65.5)	0.026
Previous stroke	158 (9.0)	2 (1.6)	48 (7.3)	65 (10.3)	43 (12.5)	0.001
Previous MI	359 (20.4)	4 (3.3)	98 (14.8)	159 (25.2)	98 (28.4)	<0.001
CABG	336 (19.1)	3 (2.4)	80 (12.1)	151 (23.9)	102 (29.6)	<0.001
PCI	344 (19.5)	6 (4.9)	76 (11.5)	153 (24.2)	109 (31.6)	<0.001
PAD	207 (11.8)	4 (3.3)	53 (8.0)	89 (14.1)	61 (17.7)	<0.001
Dyslipidemia	1250 (71.0)	47 (38.2)	404 (61.1)	511 (80.9)	288 (83.5)	<0.001
Hypertension	1586 (90.1)	99 (80.5)	585 (88.5)	576 (91.1)	326 (94.5)	<0.001
Atrial fibrillation	743 (42.2)	28 (22.8)	289 (43.7)	277 (43.8)	149 (43.2)	<0.001
COPD	291 (16.5)	4 (3.3)	85 (12.9)	101 (16.0)	101 (29.3)	<0.001
Asthma	194 (11.0)	5 (4.1)	39 (5.9)	84 (13.3)	66 (19.1)	<0.001
Diabetes mellitus	788 (44.7)	21 (17.1)	209 (31.6)	336 (53.2)	222 (64.3)	<0.001
Thyroid diseases	332 (18.9)	5 (4.1)	96 (14.5)	148 (23.4)	83 (24.1)	<0.001

*Values are* n*, (%) or median (interquartile ranges). ACE-I = angiotensin-converting enzyme inhibitor. ALT = alanine transaminase. ARB = angiotensin receptor blocker. AST = aspartate aminotransferase. BMI = body mass index. CABG = coronary artery bypass grafting. COPD = chronic obstructive pulmonary disease. DBP = diastolic blood pressure. eGFR = estimated glomerular filtration rate. HF = heart failure. LVEF = left ventricular ejection fraction. MI = myocardial infarction. NYHA = New York Heart Association. PAD = peripheral arterial disease. PCI = percutaneous coronary intervention. PLT = platelet count. QRS = Quasi Random Signal; a pattern seen in an electrocardiogram that indicates the pulses in a heart beat and their duration. SBP = systolic blood pressure. Spironolactone = medication that is primarily used to treat fluid build-up due to heart failure, liver scarring, or kidney disease. WBC = white blood cell count.*

There were no significant differences in age, sex, race, or spironolactone randomisation among the studied groups. Baseline echocardiographic data were available among 654 patients (see Supplementary Table S2 for details).

### Association of outcomes with high medication burden

**Figure 3.**
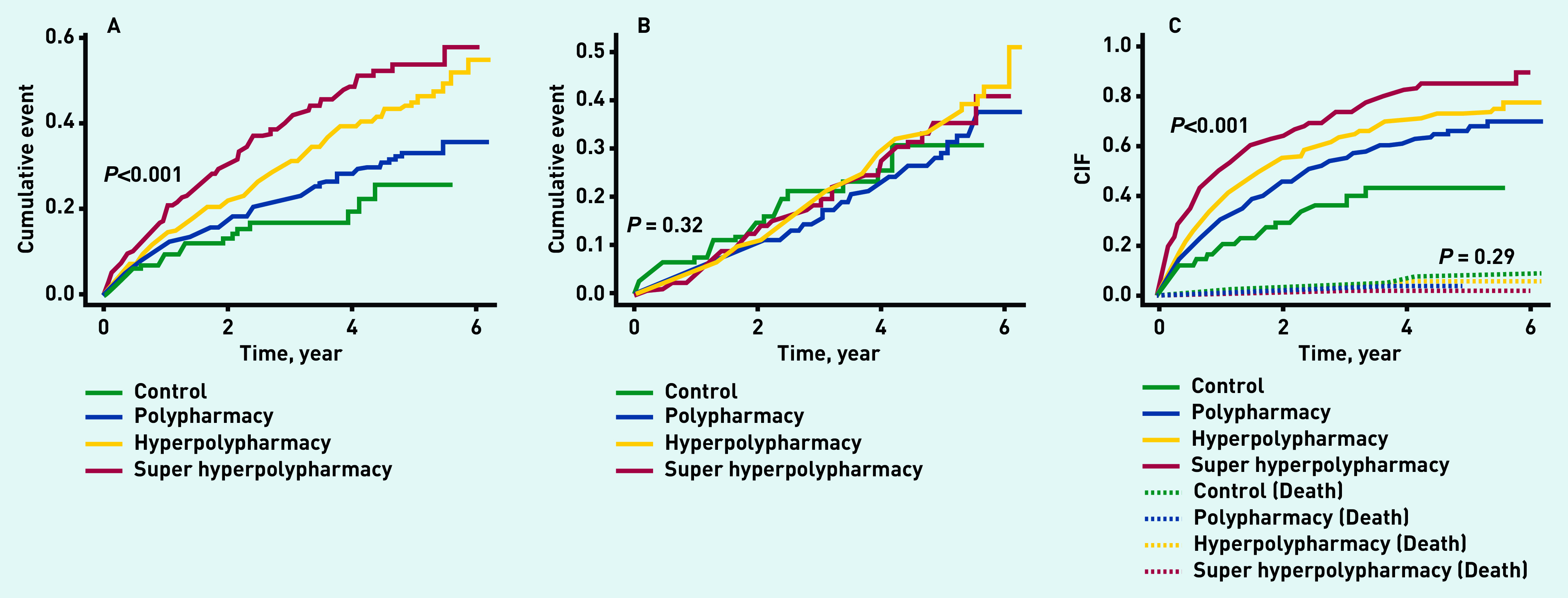
**Cumulative incidence curves. A) primary composite outcome; B) all-cause death; C) all-cause hospitalisation according to total medication burden at baseline.** **CIF = cumulative incidence function.**

The incidences of the studied outcomes are presented in Supplementary Table S3. The incidence of primary outcome was 6.3 (95% CI = 3.8 to 9.7) per 100 patient-years in patients with low medication burden, 8.7 (95% CI = 7.4 to 10.2) per 100 patient-years in patients with polypharmacy, 12.5 (95% CI = 10.9 to 14.4) per 100 patient-years in patients with hyperpolypharmacy, and 16.8 (95% CI = 14.2 to 19.9) per 100 patient-years in patients with super hyperpolypharmacy. The cumulative incidence curves of primary composite outcome, all-cause death, and all-cause hospitalisation according to total medication burden at baseline are shown in Figure 3.

Table 2 indicates both crude and adjusted HRs for the studied outcomes classified by total medication burden at baseline. After the maximised adjustment for confounders, there were no significant differences in the risk of the primary outcome, cardiovascular death, myocardial infarction, or stroke between high medication burden groups and controls.

**Table 2.** Crude and adjusted HRs for the studied outcomes by total medication burden at baseline

	**HR (95% CI)**			

	**Crude**	***P*-value**	**Adjusted^a^**	***P*-value**
**Primary outcome^b^**				
Polypharmacy versus Controls	1.40 (0.88 to 2.23)	0.16	1.05 (0.65 to 1.68)	0.85
Hyperpolypharmacy versus Controls	2.00 (1.26 to 3.16)	0.003	1.29 (0.80 to 2.07)	0.30
Super hyperpolypharmacy versus Controls	2.65 (1.66 to 4.23)	<0.001	1.37 (0.83 to 2.25)	0.22

**Cardiovascular death**				
Polypharmacy versus Controls	0.90 (0.51 to 1.60)	0.73	0.70 (0.39 to 1.25)	0.23
Hyperpolypharmacy versus Controls	0.94 (0.53 to 1.66)	0.83	0.64 (0.35 to 1.18)	0.15
Super hyperpolypharmacy versus Controls	1.08 (0.60 to 1.96)	0.80	0.66 (0.34 to 1.26)	0.21

**All-cause death**				
Polypharmacy versus Controls	0.84 (0.54 to 1.28)	0.41	0.60 (0.39 to 0.94)	0.025
Hyperpolypharmacy versus Controls	1.00 (0.65 to 1.52)	0.99	0.61 (0.39 to 0.96)	0.031
Super hyperpolypharmacy versus Controls	0.95 (0.61 to 1.49)	0.83	0.51 (0.31 to 0.83)	0.007

**HF hospitalisation^c^**				
Polypharmacy versus Controls	2.66 (1.30 to 5.44)	0.007	2.12 (1.02 to 4.40)	0.043
Hyperpolypharmacy versus Controls	4.25 (2.09 to 8.60)	<0.001	2.83 (1.37 to 5.86)	0.005
Super hyperpolypharmacy versus Controls	5.65 (2.77 to 11.55)	<0.001	3.00 (1.43 to 6.31)	0.004

**All-cause hospitalisation^c^**				
Polypharmacy versus Controls	1.75 (1.27 to 2.42)	0.001	1.51 (1.09 to 2.10)	0.014
Hyperpolypharmacy versus Controls	2.26 (1.64 to 3.11)	<0.001	1.81 (1.29 to 2.53)	0.001
Super hyperpolypharmacy versus Controls	3.17 (2.28 to 4.42)	<0.001	2.29 (1.61 to 3.27)	<0.001

**Myocardial infarction^c^**				
Polypharmacy versus Controls	1.81 (0.55 to 5.97)	0.33	1.31 (0.39 to 4.40)	0.67
Hyperpolypharmacy versus Controls	1.88 (0.57 to 6.19)	0.30	1.11 (0.33 to 3.80)	0.86
Super hyperpolypharmacy versus Controls	3.37 (0.99 to 11.13)	0.05	1.74 (0.46 to 6.51)	0.41

**Stroke^c^**				
Polypharmacy versus Controls	1.82 (0.55 to 6.01)	0.33	1.65 (0.49 to 5.50)	0.42
Hyperpolypharmacy versus Controls	1.40 (0.42 to 4.70)	0.58	1.25 (0.35 to 4.39)	0.73
Super hyperpolypharmacy versus Controls	2.10 (0.62 to 7.16)	0.23	1.92 (0.52 to 7.07)	0.33

a Adjusted for age, sex, race, diastolic blood pressure, smoking status, New York Heart Association functional class, hemoglobin, serum creatinine, history of HF hospitalisation, arterial disease, diabetes mellitus, chronic obstructive pulmonary disease.

b Primary outcome was composite of cardiovascular disease death, aborted cardiac arrest, or HF hospitalisation.

c Using competing risks regression. HF = heart failure. HR = hazard ratio.

Compared with controls, high medication burden was associated with a reduced risk of all-cause death (polypharmacy: HR = 0.60, 95% CI = 0.39 to 0.94; hyperpolypharmacy: HR = 0.61, 95% CI = 0.39 to 0.96; super hyperpolypharmacy: HR = 0.51, 95% CI = 0.31 to 0.83), but had increased risks of HF hospitalisation (polypharmacy: HR = 2.12, 95% CI = 1.02 to 4.40; hyperpolypharmacy: HR = 2.83, 95% CI = 1.37 to 5.86; super hyperpolypharmacy: HR = 3.00, 95% CI = 1.43 to 6.31) and all-cause hospitalisation (polypharmacy: HR = 1.51, 95% CI = 1.09 to 2.10; hyperpolypharmacy: HR = 1.81, 95% CI = 1.29 to 2.53; super hyperpolypharmacy: HR = 2.29, 95% CI = 1.61 to 3.27).

The subgroup analysis based on LVEF illustrated statistically insignificant interaction effects for all-cause death, HF hospitalisation, and all-cause hospitalisation (*P*_interaction_ >0.05; see Supplementary Table S4 for details).

### Predictors of high medication burden

Supplementary Table S5 shows the univariable models for the risk factors of using ≥5 medications, ≥10 medications, and ≥15 medications. There was no independent predictor of using ≥5 medications demonstrated in the multivariable models. The predictors of using ≥10 medications and ≥15 medications derived from the multivariable regression models are summarised in Table 3. It was found that dyslipidemia (risk ratio [RR] = 1.38, 95% CI = 1.17 to 1.62), angina pectoris (RR = 1.23, 95% CI = 1.05 to 1.44), thyroid disease (RR = 1.29, 95% CI = 1.05 to 1.59), and DM (RR = 1.24, 95% CI = 1.05 to 1.46) were independently associated with using ≥10 medications, while angina pectoris (RR = 1.44, 95% CI = 1.19 to 1.75), DBP <80mm Hg (RR = 1.35, 95% CI = 1.06 to 1.73), NYHA functional class III/IV (RR = 1.22, 95% CI = 1.02 to 1.46), DM (RR = 1.30, 95% CI = 1.26 to 1.35), COPD (RR = 1.38, 95% CI = 1.12 to 1.71), and anaemia (RR = 1.31, 95% CI = 1.10 to 1.57) were independently associated with using ≥15 medications.

**Table 3.** Predictors of using ≥10 medications and ≥15 medications in the multivariable models

	**Risk ratio^a^ (95% CI)**	***P-*value**
**≥10 medications**		
Dyslipidemia	1.38 (1.17 to 1.62)	<0.001
Angina pectoris	1.23 (1.05 to 1.44)	0.010
Thyroid disease	1.29 (1.05 to 1.59)	0.015
Diabetes mellitus	1.24 (1.05 to 1.46)	0.009

**≥15 medications**		
Angina pectoris	1.44 (1.19 to 1.75)	<0.001
DBP < 80mmHg	1.35 (1.06 to 1.73)	0.015
NYHA functional class III and IV	1.22 (1.02 to 1.46)	0.029
Diabetes mellitus	1.30 (1.26 to 1.35)	<0.001
COPD	1.38 (1.12 to 1.71)	0.003
Anaemia	1.31 (1.10 to 1.57)	0.003

a Adjusted for the predictors that showed significance in univariable models (see Supplementary Table S4 for details). COPD = chronic obstructive pulmonary disease. DBP = diastolic blood pressure. NYHA = New York Heart Association.

## DISCUSSION

### Summary

In the present study, based on data from the TOPCAT trial, the results indicated that:

- the prevalence of polypharmacy, hyperpolypharmacy, and super hyperpolypharmacy were 38%, 36%, and 19%, respectively;
- high medication burden was associated with a reduced risk of all-cause death, but with increased risks of HF hospitalisation and all-cause hospitalisation; and
- dyslipidemia, angina pectoris, DBP <80 mmHg, NYHA functional class III/IV, thyroid diseases, DM, COPD, and anaemia were independent predictors of high medication burden in patients with HFpEF.

### Strengths and limitations

To the authors’ knowledge, this study is the first to examine the relationship between high medication burden and the prognostic outcomes in patients with HFpEF. The topic is clinically relevant because polypharmacy is an increasing clinical situation, frequently considered as an inevitable circumstance, and its potential prognostic impact could be underestimated. This present article focused on a single phenotype of HF, and carefully stratified the medication burden. Thus, more precise associations between clinical outcomes and each level of medication burden were demonstrated. Several limitations might influence the validity of the present findings. First, the study is a retrospective analysis of the TOPCAT trial; and thus, the residual confounders from unmeasured factors might affect the findings. Second, the study defined high medication burden based on the published studies due to there being no universally accepted definitions of them. Third, the study did not examine which drug category was seemingly the most common type associated with adverse outcomes. Also, the effects of drug dosages on outcomes were aborted to be assessed, though the drug dosages by total medication burden at baseline have been shown in Supplementary Table S6.

Finally, the inappropriate use of drugs might negatively affect patients’ prognosis. Although several confounding factors were adjusted, the study could not distinguish whether the drugs were used appropriately in the present analysis.

### Comparison with existing literature

As populations continue to age and as the prevalence of comorbidity and multimorbidity grows, patients with HF may increasingly require an elaborate therapeutic scheme with multiple medications. The prevalence of high medication burden in patients with HF is presumed to increase over time, but varies across different studies. Data from the National Health and Nutrition Examination Survey suggested an increasing average number of medications from 4.1 to 6.4 prescriptions per patient with HF over the past two decades.^19^ In another study, the median number of medications in community-dwelling patients with HF was 11, and 12% of patients received >20 drugs.^5^ A large cross-sectional study has separately investigated the prevalence of comorbidities and polypharmacy in HF patients due to left ventricular systolic dysfunction.^20^

Previous studies indicated that the prevalence of patients (mixed population of HFrEF and HFpEF) with a number of prescriptions ≥5 or ≥10 was 74%^21^ or 26%,^4^ respectively. In the present study, the prevalence rates of ≥5 medications and ≥10 medications were 93% and 55%, respectively, in patients with HFpEF. Seemingly, this study had a greater prevalence of high medication burden, probably because of the higher comorbidity burden in the HFpEF population compared with patients with HFrEF.^22^ Moreover, high medication burden was associated with non-cardiovascular medications, suggesting that comorbidities like obesity, DM, and chronic lung disease are on the rise.^23^

Previous studies found that the number of comorbidities, ≥10 contacts with ambulatory healthcare services, ≥3 hospitalisations, low household income, low educational status,^4^ and cognitive impairment,^4^ but not functional impairment,^4^^,^^21^ were independently related with hyperpolypharmacy in patients with HF. Apart from comorbidities, this study found that DBP <80 mmHg was likely an underlying predictor of super hyperpolypharmacy in patients with HFpEF, which was consistent with the findings of previous studies suggesting that a low DBP elevated the risks of adverse outcomes in patients with HFpEF,^24^ and that the relationship between decreasing DBP and increased risk of hospitalisation was linear.^25^

In patients with HF, high medication burden could lead to poor medication adherence and persistence,^7^ drug-drug interactions,^8^ underuse of effective treatment, inappropriate drug prescription, adverse drug-related effects,^9^ and multiple taste disturbances.^26^

High medication burden is common among older people with multiple comorbidities who usually have poorer medication compliance than young patients. However, the current study revealed no significant difference in age between patients stratified under the different polypharmacy groups. High medication burden was found to be associated with increased risks of HF hospitalisation and all-cause hospitalisation herein.

Understandably, side effects induced by high medication burden would account for a significant proportion of hospitalisations.^2^ In addition, patients with HF are often concurrently prescribed with HF-exacerbating medications before hospital discharge,^27^ which may consequently result in higher risk of re-hospitalisation.

Nevertheless, high medication burden did not significantly impact mortality in patients with HFpEF in this retrospective analysis.

### Implications for research and practice

Based on these data, a high medication burden could substantially exacerbate the risk of hospitalisation. However, whether this reflects the aforementioned or other unknown factors through which high medication burden would impact the outcomes of HF patients needs further investigation. It is hard to assess which drug might not be beneficial and thus could be ceased,^28^ because whether medications could be safely withdrawn in patients with HF is still controversial.^29^^,^^30^ Therefore, choosing the optimal drugs for patients with HF would be a challenge for clinicians.

In this scenario, proper management of HF-related medications is a key priority. It is necessary to implement a multidisciplinary team approach involving both clinicians and pharmacists in medical practice to improve the therapeutic and socioeconomic outcomes of high medication burden.^31^

Medication therapy management (MTM) services are designed to optimise the use of medications. MTM interventions might improve the occurrence of high healthcare costs, medication non-adherence, inappropriate drug prescription, and adverse drug-related effects,^32^ but still have insufficient evidence on long-term clinical outcomes.

More research is still required to further enhance the comprehensive management in HF.
